# Supplementary material for: Pathologic Proteolytic Processing of N-Cadherin as a Marker of Human Fibrotic Disease
Source: Cells. 2022 Jan 4;11(1):156. doi: 10.3390/cells11010156 (PMC8750447; doi:10.3390/cells11010156)
Supplement: Supplementary file 1 [file cells-11-00156-s001.zip › cells-1522206-supplementary.pdf]

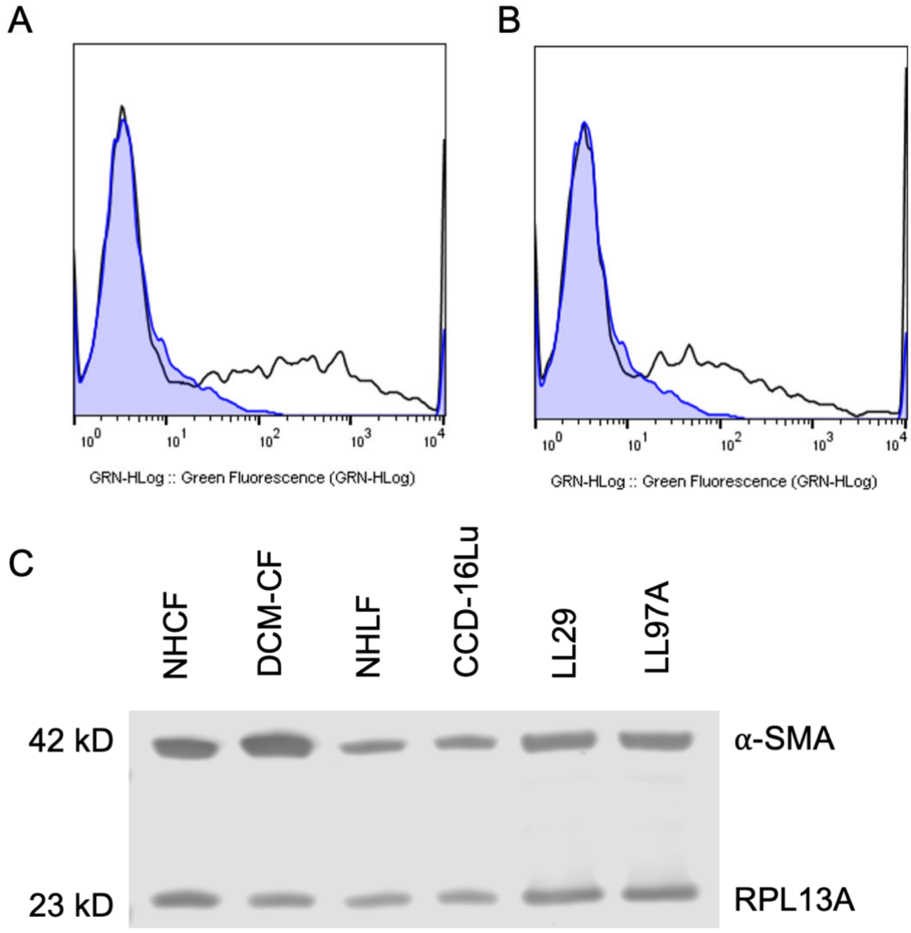
A
B
GRN-HLog :: Green Fluorescence (GRN-HLog)
GRN-HLog :: Green Fluorescence (GRN-HLog)
C
NHCF
DCM-CF
NHLF
CCD-16Lu
LL29
LL97A
42 kD
23 kD
α-SMA
RPL13A

Supplementary Figure S1. Validation of myofibroblast phenotype of explant tissue derived cells and cell lines. (A) Rabbit IgG isotype control (blue shaded) was compared to α-SMA antibody (unshaded); Chi-squared = 102.0; SE Dymax % Positive = 32.1. (B) Rabbit IgG isotype control (blue shaded) was compared to type I collagen antibody (unshaded); Chi-squared = 142.0; SE Dymax % Positive = 36.0. (C) Total cell lysates were immunoblotted from each cell line to determine α-SMA protein expression and confirm myofibroblast phenotype.

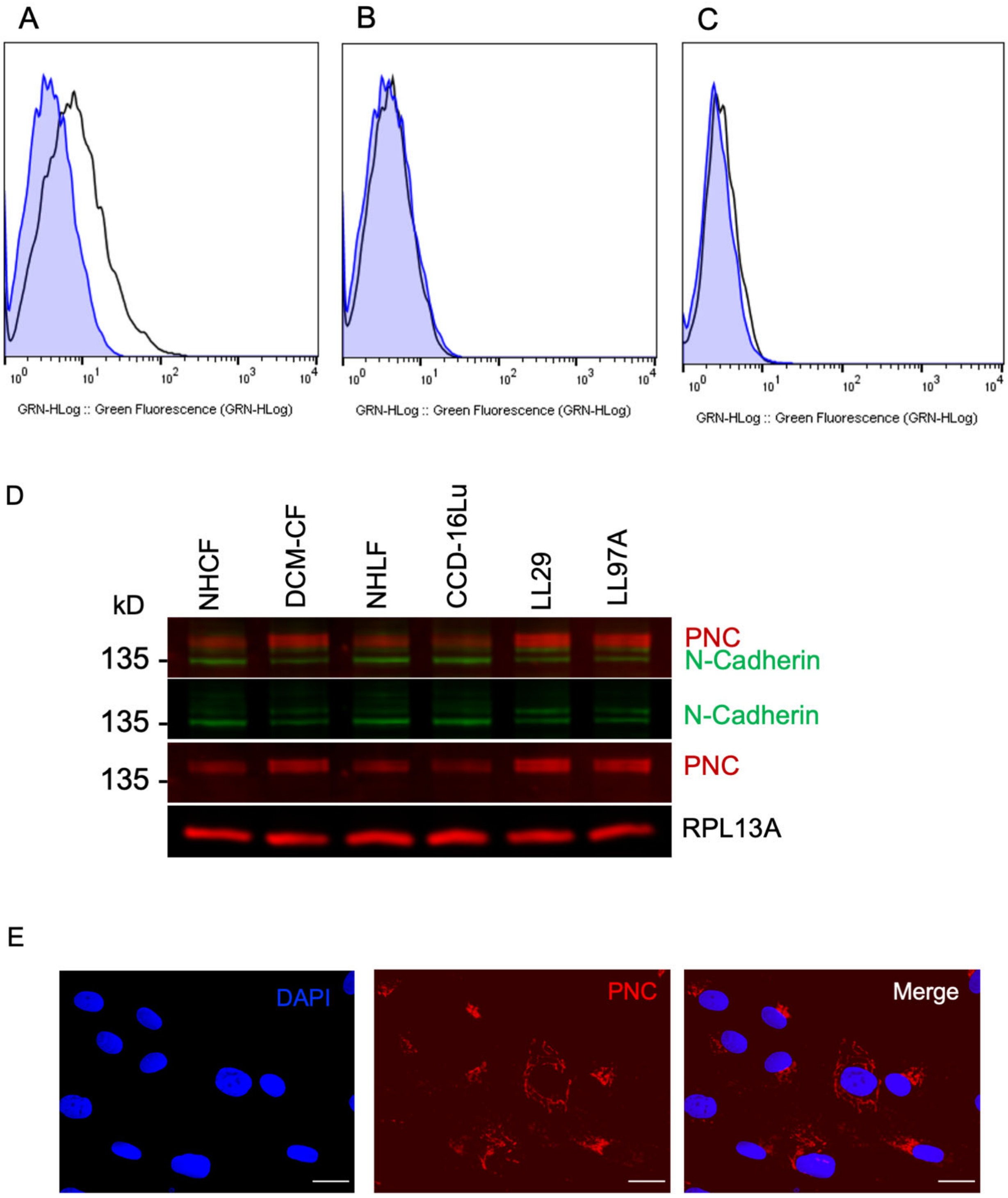

A
B
C
GRN-HLog :: Green Fluorescence (GRN-HLog)
GRN-HLog :: Green Fluorescence (GRN-HLog)
GRN-HLog :: Green Fluorescence (GRN-HLog)
D
kD
NHCF
DCM-CF
NHLF
CCD-16Lu
LL29
LL97A
135
135
135
PNC
N-Cadherin
N-Cadherin
PNC
RPL13A
E
DAPI
PNC
Merge

Supplementary Figure S2. Validation of N-cadherin reagent specificity. CCD-16Lu were analyzed by flow cytometry following the flow cytometry protocol in the text to confirm no cross reactivity between α-PNC mAb and mature N-cadherin. (A) Analysis comparing mouse IgG1 isotype control (blue shaded) was compared to antibody to mature N-cadherin (unshaded); Chi-squared = 301.4; SE Dymax % Positive = 43.2. (B) Mouse IgG1 isotype control (blue shaded) was compared to antibody m-α-PNC mAb 10A10 (unshaded); Chi-squared = 0; SE Dymax % Positive = 10.9. (C) Human IgG4 isotype control (blue shaded) was compared to antibody h-α-PNC mAb HC5LC4 (unshaded); Chi-squared = 1.2; SE Dymax % Positive = 14.7 . All antibodies were used at 5µg/mL. (D) Total cell lysate from each cell line was immunoblotted for mature N-cadherin, PNC and RPL13A for a loading control. (E) Representative image of permeabilized and immunostained myofibroblasts with perinuclear PNC expression. Scale bars represent 25µm.

A

	k_d (s-1)
HC1 LC1	<0.0000001
HC3 LC3	<0.0000001
HC5 LC4	0.0000367
HC4 LC4	0.0000688
HC1 LC5	0.0000975
HC5 LC5	0.0001310
HC3 LC4	0.0001360
HC1 LC3	0.0001470
HC3 LC2	0.0001550
HC5 LC2	0.0001740
HC2 LC3	0.0001810
HC1 LC4	0.0002240
HC2 LC4	0.0002260
HC2 LC2	0.0002820
HC2 LC5	0.0002830
HC1 LC2	0.0003120
HC5 LC1	0.0003220
HC5 LC3	0.0003430
HC2 LC1	0.0003920
HC3 LC1	0.0004260
HC0 LC0	0.0004810
HC3 LC5	0.0007610

B

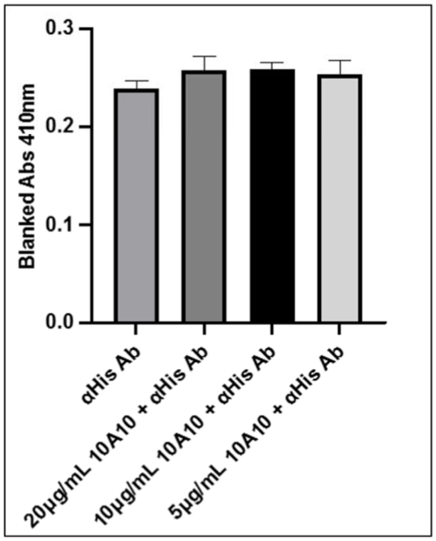

Supplementary Figure S3. (A) Humanized α-PNC mAb variants ranked by dissociation rate. A total of 22 humanized PNC monoclonal antibody variants produced from the sequence of murine IgG1 clone 10A10. HC0 LC0 is an intermediate chimeric IgG4 mAb containing identical variable and CDR regions of murine 10A10. Variants highlighted in yellow did not fit well to a 1:1 binding model and were not pursued further. (B) Recombinant prodomain of N-cadherin was immobilized onto a high binding ELISA plate, blocked, then incubated with anti-his-tag antibody alone or with varying concentrations of anti-PNC antibody clone 10A10. Biotinylated anti-his-tag antibody was then detected using streptavidin-HRP.
